# 6-Shogaol Suppresses 2-Amino-1-Methyl-6-Phenylimidazo [4,5-b] Pyridine (PhIP)-Induced Human 786-O Renal Cell Carcinoma Osteoclastogenic Activity and Metastatic Potential

**DOI:** 10.3390/nu11102306

**Published:** 2019-09-28

**Authors:** I-Jeng Yeh, Szu-Chia Chen, Meng-Chi Yen, Yen-Hung Wu, Chih-Hsing Hung, Po-Lin Kuo

**Affiliations:** 1 Graduate Institute of Clinical Medicine, College of Medicine, Kaohsiung Medical University, Kaohsiung 807, Taiwan; ijengyeh@hotmail.com (I.-J.Y.); scarchenone@yahoo.com.tw (S.-C.C.); yohoco@gmail.com (M.-C.Y.); blan32705@hotmail.com (Y.-H.W.); 2 Department of Emergency Medicine, Kaohsiung Medical University Hospital, Kaohsiung 807, Taiwan; 3 Faculty of Medicine, College of Medicine, Kaohsiung Medical University, Kaohsiung 807, Taiwan; 4 Division of Nephrology, Department of Internal Medicine, Kaohsiung Medical University Hospital, Kaohsiung 807, Taiwan; 5 Department of Internal Medicine, Kaohsiung Municipal Hsiao-Kang Hospital, Kaohsiung 812, Taiwan; 6 Department of Pediatrics, Kaohsiung Municipal Hsiao-Kang Hospital, Kaohsiung 812, Taiwan; 7 Department of Pediatrics, Faculty of Medicine, College of Medicine Kaohsiung Medical University, Kaohsiung 807, Taiwan; 8 Research Center for Environmental Medicine, Kaohsiung Medical University, Kaohsiung, Taiwan; 9 Institute of Medical Science and Technology, National Sun Yat-Sen University, Kaohsiung 804, Taiwan; 10 Center for Cancer Research, Kaohsiung Medical University, Kaohsiung 807, Taiwan

**Keywords:** Ginger, renal cell carcinoma, PhIP, PTHrP, osteoclastogenesis

## Abstract

2-Amino-1-methyl-6-phenylimidazo [4,5-b]pyridine (PhIP) which can be detected in processed meats and red meats, is a potential carcinogen for renal cell carcinoma (RCC). Approximately 30% of patients with metastatic RCC have bone metastases, and the prognosis of RCC with bone metastases is poor. Thus, the aim of the present study was to investigate whether PhIP induced bone metastases and to develop novel therapeutic agents. Our data revealed that PhIP pre-treatment increased the production of parathyroid hormone-related protein (PTHrP) in human 786-O renal cell carcinoma cells. Subsequently, the cultures of human osteoblasts with PhIP-stimulated condition medium of 786-O increased the expression of the macrophage colony-stimulating factor (M-CSF) and receptor activator of nuclear factor kappa-B ligand (RANKL), and decreased the expression of osteoprotegerin (OPG). In addition, PhIP-mediated PTHrP up-regulated as well as increased IL-8 secretion in 786-O cells, and then contributed to 786-O-mediated bone resorption. Furthermore, 6-shogaol, which is an active ingredient in ginger, showed suppressive effects on PhIP-mediated bone resorption. In summary, this is the first study to demonstrate that PhIP pre-treatment increases the stimulatory effect of human renal cell carcinoma 786-O on osteoclastogenesis activity directly by PTHrP. In addition, 6-shogaol treatment reverses PhIP-mediated bone resorption. It suggests that 6-shogaol treatment results in bone resorption activity in the RCC model in vitro.

## 1. Introduction

Ginger (Zingiber officinale) is distributed throughout the tropical and subtropical regions [1]. It is used not only in cooking but as a medical plant in South East Asia and other countries [1,2], and since ancient times, it has been applied to treat a range of diseases [3,4]. Carbohydrates (50–70%), lipids (3–8%), terpenes, and phenolic compounds are four major constituents of ginger [5]. Zingiberene, β-bisabolene, α-farnesene, β-sesquiphellandrene, and α-curcumene are major terpene components, and gingerol, paradols, and shogaols are major components in phenolic compounds in ginger [6]. 6-shogaol is also a major compound of shogaols in fresh ginger (average content is 133.2 mg/kg) [2], and as an active ingredient, has demonstrated its anti-inflammatory and anti-cancer effects in various in vitro and in vivo experimental models [7,8,9,10,11,12,13,14,15]. Consequently, the intake of ginger extract is considered to be beneficial to health.

2-Amino-1-methyl-6-phenylimidazo [4,5-b]pyridine (PhIP) is an abundant heterocyclic aromatic amine that can be found in processed meats and red meats [16]. The International Agency for Research on Cancer has classified processed meats and red meats as carcinogenic and probably carcinogenic to humans respectively [17]. PhIP causes prostate, colon and mammary cancers in rats [18,19]. Additionally, the consumption of PhIP is a potential risk factor for the development of breast cancer in humans [20], while the intake of PhIP and benzo(a)pyrene is associated with an elevated risk of renal cell carcinoma (RCC) [21,22].

RCC is a kidney cancer arising from proximal convoluted tubules [23] and is the most common type of kidney cancer in adults [24], typically metastasizing to the lungs, lymph nodes, liver, adrenal glands, brain and bone [25,26]. According to clinical data, the 5-year survival rate of patients with metastatic RCC is lower than 10% [27]. Approximately one-third of patients with metastatic RCC have bone metastasis [28], which is currently classified as osteolytic, osteoblastic, and mixed bone metastases [29]. Similar to breast cancer, osteolytic lesions are more common than other types of bone metastases in RCC [29,30].

Parathyroid hormone-related protein (PTHrP) is a polyprotein involved in the autocrine regulation of cell growth in RCC cell lines [31,32]. By blocking PTHrP with specific antibodies against the PTHrP/PTHrP receptor induces cell death in vitro and in a xenograft tumor model [33]. In osteolytic metastasis of breast cancer, PTHrP produced from cancer cells stimulates the formation of osteoclast [34]. Furthermore, PTHrP increases the proliferation of osteoblast progenitor cells and induces early osteoblast differentiation [35]. This evidence suggests that PTHrP might also interact with osteoblast and osteoclast cells and promote bone metastasis processes of RCC. Since the PhIP is a potential carcinogen within processed red meat, this study aimed to evaluate whether PhIP-stimulated RCC cells affect osteoclastogenesis activity via the regulation of PTHrP. In addition, the anti-metastasis activity of 6-shogaol, a bioactive component in ginger, was evaluated in the present study.

## 2. Materials and Methods

### 2.1. Chemicals

All chemicals including 6-Shogaol, dimethyl sulfoxide (DMSO), and 2-Amino-1-methyl- 6-phenylimidazo [4,5-b]pyridine (PhIP), were purchased from Sigma-Aldrich (St Louis, MO, USA). The stock solution of PhIP was dissolved in 1 mL of DMSO at the concentration of 20 mM and the stock solution of 6-Shogaol was dissolved in 10 mL of DMSO at the concentration of 2 mM. All stock of PhIP and 6-Shogaol were divided into 10 identical aliquots and stored at −20°C.

### 2.2. Cell Culture and Conditioned Medium

786-O is a human renal cell carcinoma cell line. This cell line was obtained from the Bioresource Collection and Research Center (BCRC 60243) (Hsinchu, Taiwan). Human primary osteoblasts were obtained from Lonza (Walkersville, MD, USA). Further, 786-O cells were cultured in RPMI-1640 (Gibco-BRL, Gaithersburg, MD, USA) medium that was supplied with 10% fetal bovine serum (FBS) (Gibco-BRL, Gaithersburg, MD, USA), and human primary osteoblasts were cultured in an osteoblast growth medium (OBM) (Lonza Walkersville, MD, USA). Before collecting the condition media (CM) of 786-O, 786-O cells (1 × 10^6^ /100 mm dish) were seeded and treated with 20 μM PhIP treatment for 6 h. Subsequently, the medium was replaced and cultured for 24 h, then the supernatant was harvested and filtered by 0.22 μm filter, and finally defined as the CM of 786-O.

### 2.3. Measurement of Secreted Factors

The levels of OPG, M-CSF, RANKL and IL-8 were measured via DuoSet enzyme-linked immuno-sorbent assay from R&D Systems (Minneapolis, MN, USA). The levels of Parathyroid hormone-related protein (PTHrP) were also determined by an ELISA kit from Abnova Corporation (Taipei, Taiwan).

### 2.4. Isolation of CD14^+^ Monocytes and Osteoclast Differentiation

The peripheral blood samples were collected from five healthy donors after informed consent was obtained. The Institutional Review Board of Kaohsiung Medical University Hospital approved the study protocol and all of the participants provided written informed consent in accordance with the Declaration of Helsinki. The mononuclear cells were isolated by Ficoll-Hypaque gradient (GE Healthcare Bio-Sciences, Little Chalfont, Buckinghamshire, UK) according to the manufacturer’s instructions. The CD14^+^ monocytes were then isolated from these mononuclear cells via human CD14 MicroBeads (Miltenyi Biotec Ltd., Bergisch Gladbach, Germany). In order to generate osteoclasts, CD14^+^ monocytes were cultured for 14–21 days in a medium supplemented with 100 ng/mL M-CSF, 50 ng/mL RANKL (R&D Systems, Minneapolis, MN, USA), and 20% of the condition medium. During the period of osteoclast differentiation, the medium was replaced every five days with fresh medium containing M-CSF and RANKL. As tartrate-resistant acid phosphate (TRAP) activity is a marker of osteoclast [36], the Acid Phosphatase, Leukocyte (TRAP) Kit (Sigma-Aldrich, St. Louis, MO, USA) was used for confirming osteoclast differentiation. The cell number and nuclei per cells were counted under light microscopy. In this study, TRAP-positive was defined when the cells had three or more nuclei. However, a 48-well plate bone resorption assay (Cosmo Bio Co., Ltd., Tokyo, Japan) was used for determining the bone resorption activity, and the pit area was determined by AlphaEase FC Software (version 6.0.0, Alpha Innotech Corporation, San Leandro, CA, USA).

### 2.5. Real-Time Polymerase Chain Reaction (qRT-PCR)

The total RNA was extracted via the TRIzol reagent (Invitrogen, Carlsbad, CA, USA) and cDNA was reverse-transcripted by oligo-dT primer and PrimeScript RT Reagent Kit (Takara, Shiga, Japan). To determine the mRNA expression, the PCR reaction was performed via 2× SYBR Green PCR Master Mix (Applied Biosystems, Foster City, CA, USA) according to the manufacturer’s instructions on PCR instruments (StepOne-Plus, Applied Biosystems, Foster City, CA, USA). The program of PCR was 95 °C for 10 min, and then for 40 cycles at 95 °C for 15 s and 60 °C for 1 min. The gene expression was normalized to glyceraldehyde-3-phosphate dehydrogenase and the relative expression was presented using the 2^−△△CT^ method [37].

### 2.6. PTHrP Knockdown

The 786-O renal cell carcinoma cells were transfected with 20 nM non-target or PTHrP siRNAs pooled by DharmFECT 4 reagents (Dharmacon, Lafayette, CO, USA). After 24 h of transfection, the medium was changed to fresh medium and the transfected cells were then treated with PhIP. The knockdown efficiency of PTHrP was measured by qRT-PCR.

### 2.7. Statistical Analysis

The bar graphs were expressed as the means ± standard deviation (SD) and each value was obtained from three independent experiments. The statistical comparisons of the results were made using the Student’s *t*-test (between 2 samples) or the analysis of variance (ANOVA, more than 3 samples). The significant differences were considered when *P* value was <0.05.

## 3. Results

### 3.1. 2-Amino-1-Methyl-6-Phenylimidazo [4,5-b]pyridine (PhIP) Induced Parathyroid Hormone-Related Protein (PTHrP) Secretion In Human 786-O Renal Cell Carcinoma Cells

**Figure 1:**
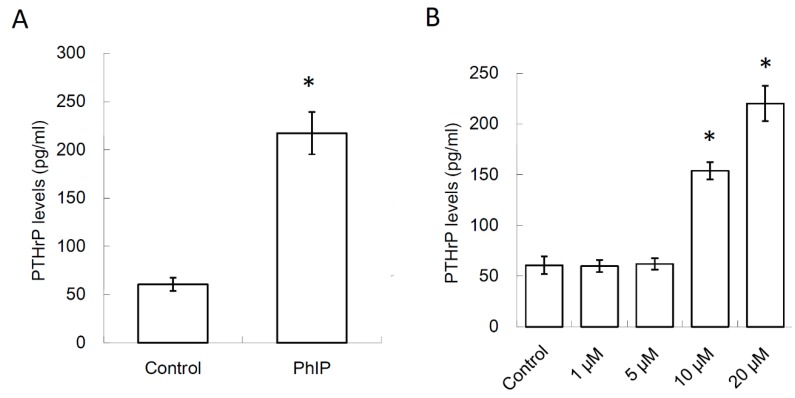
The effect of 2-Amino-1-methyl-6-phenylimidazo [4,5-b]pyridine (PhIP) on parathyroid hormone-related protein (PTHrP)-secreted renal cell carcinoma cell 786-O. The PTHrP levels in the condition medium (CM) of 786-O were detected by ELISA. (**A**) The PTHrP levels of 786-O CM after 20 μM PhIP pre-treatment. (**B**) The PTHrP levels of 786-O cells after different dose of PhIP pre-treatment. Each value was the mean ± standard deviation (SD) of three independent experiments. * *P* < 0.05, significant difference between the control and test groups.

Whether the PhIP-stimulated RCC cells affected the osteoclastogenesis activity was firstly investigated. As the effect of PhIP has been evaluated at the concentration of 20 μM in prostate cancer cells and hepatoma cells [38,39], the same dosage of PhIP was selected in the following experiments. Human renal cell carcinoma, 786-O, was treated with 0.1% DMSO (control) or PhIP 20 μM for 6 h. After removing PhIP containing the medium and culturing for 24 h, the condition medium (CM) of 786-O cells was harvested. As shown in Figure 1A, the PTHrP levels in CM of PhIP-treated 786-O was significantly higher than that in the CM of the controls. In addition, our results revealed that pre-treatment with different concentrations of PhIP increased PTHrP production in 786-O cells in a dose-dependent manner (Figure 1B).

### 3.2. Conditioned Medium (CM) of PhIP-Treated 786-O Increased Receptor Activator of Nuclear Factor Kappa-B Ligand (RANKL) and Macrophage Colony-Stimulating Factor (M-CSF) Expression, and Decreased Osteoprotegerin (OPG) Expression in Osteoblasts

**Figure 2:**
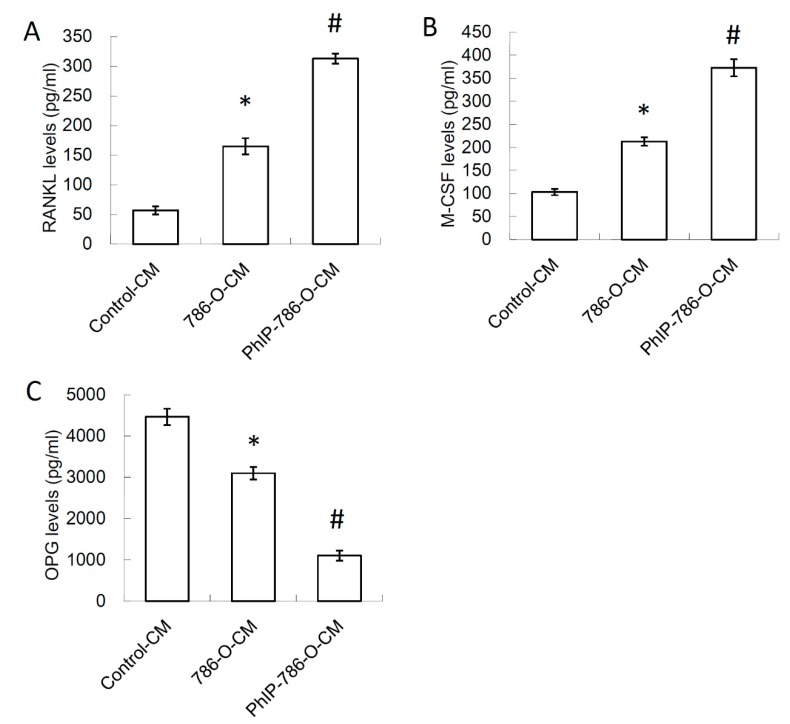
The effect of PhIP-786-O-CM on osteoblast. CM from vehicle control (0.1% DMSO)-treated and 20 μM PhIP-treated 786-O was defined as 786-O-CM and PhIP-786-O-CM. Fresh medium without culturing CM of 786-O was defined as the control-CM. The human osteoblasts were cultured with various CMs for 24 h. In the supernatants of the osteoblast-cultured media, the levels of (**A**) receptor activator of nuclear factor kappa-B ligand (RANKL) and (**B**) macrophage colony-stimulating factor (M-CSF) osteoprotegerin, and (**C**) osteoprotegerin (OPG) are shown, while bar graphs are shown as the mean ± SD of three independent experiments. * Significant difference with the control-CM treatment, # Significant difference with 786-O-CM treatment, *P* < 0.05.

Previous studies have demonstrated that PTHrP enhances osteoclastogenesis by affecting the expression of the osteoclastogenesis activator (receptor activator of nuclear factor kappa-B ligand [RANKL] and macrophage colony-stimulating factor [M-CSF]) and inhibitor (osteoprotegerin, OPG) secreted by osteoblasts [40,41]. To determine whether PhIP-induced secretory factors of 786-O affected the secretion of RANKL, M-CSF, and OPG in osteoblasts, the CM of 786-O was added to human osteoblasts. The CM of 0.1% DMSO or 20 μM PhIP treatment was defined as 786-O-CM, and PhIP-786-O-CM respectively. The fresh medium without culturing 786-O was defined as the control-CM. The secretion of RANKL and M-CSF in human osteoblasts was induced by 786-O-CM and further enhanced by PhIP-786-O-CM (Figure 2A,B). In contrast, 786-O-CM decreased the OPG expression in osteoblasts and this inhibitory effect of renal cell carcinoma in osteoblasts worsened when renal cell carcinoma was exposed to PhIP (Figure 2C).

### 3.3. PhIP Increased Human 786-O Renal Cell Carcinoma Cell-Mediated Osteoclastogenesis and Bone Resorption

**Figure 3:**
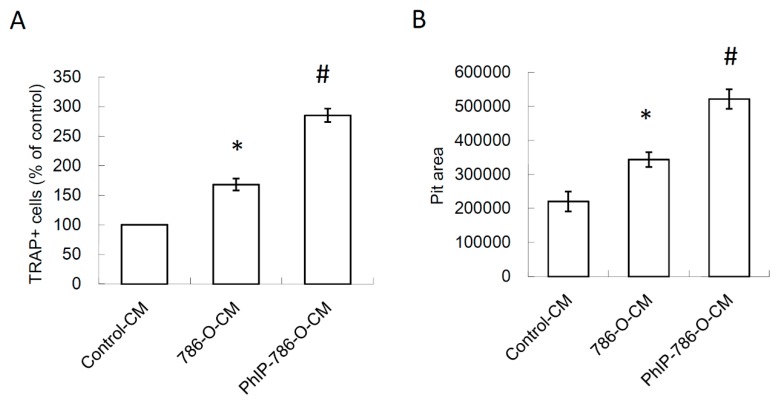
PhIP reinforced the stimulatory effect of human 786-O renal cell carcinoma on osteoclastogenesis and bone resorption on CD14^+^ monocyte-differentiated osteoclasts. (**A**) The TRAP-positive cells in CD14^+^ monocyte-differentiated cells after different CM treatment. (**B**) Bone resorption activity. Each value is shown as the mean ± SD of three independent experiments. * Significant difference with control-CM treatment, # Significant difference with 786-O-CM treatment, *P* < 0.05.

The effect of PhIP on renal cell carcinoma-mediated osteoclastogenesis on CD14^+^ monocyte-differentiated osteoclasts was further assessed. The results revealed that 786-O-CM increased osteoclastogenesis and this effect was reinforced when 786-O cells were pre-treated with PhIP (Figure 3A). The PhIP pre-treatment also further enhanced osteoclast bone resorption activity (Figure 3B).

### 3.4. PTHrP/IL-8 Autocrine Loop was Involved in the Stimulation of PhIP on Renal Cell Carcinoma-Mediated Osteoclastogenesis

**Figure 4:**
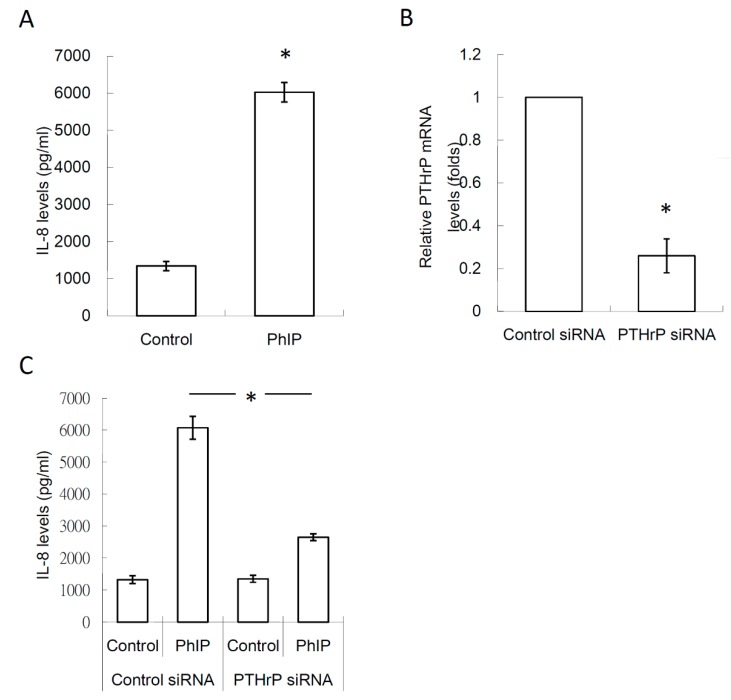
PhIP increased IL-8 production through PTHrP-mediated mechanism in 786-O. (**A**) The levels of IL-8 levels in medium of 786-O cells after vehicle control or PhIP (20 μM) treatment for 24 h. (**B**) The efficiency of PTHrP siRNA was determined via qRT-PCR. (**C**) The levels of IL-8 levels in siRNA-transfected 786-O cells. Each value is the mean ± SD of three independent experiments. * *P* < 0.05, or significant difference between the control and test groups.

Since PTHrP reportedly increased the cancer cell expression of IL-8 [42], this study evaluated whether PhIP increased the inductive effect of 786-O cells on osteoclastogenesis by targeting the PTHrP/IL-8 loop. In Figure 4A, the higher levels of IL-8 in PhIP-786-O-CM were detected when compared to the 786-O-CM control. To confirm the role of PTHrP on the up-regulation of IL-8 induced by PhIP, 786-O cells were transfected with PTHrP siRNA, and this treatment decreased the PTHrP mRNA expression in 786-O cells by 74% (Figure 4B). Silencing PTHrP in 786-O cells decreased IL-8 production in 786-O cells (Figure 4C).

### 3.5. 6-Shogaol Suppressed PhIP-Mediated Bone Resorption

**Figure 5:**
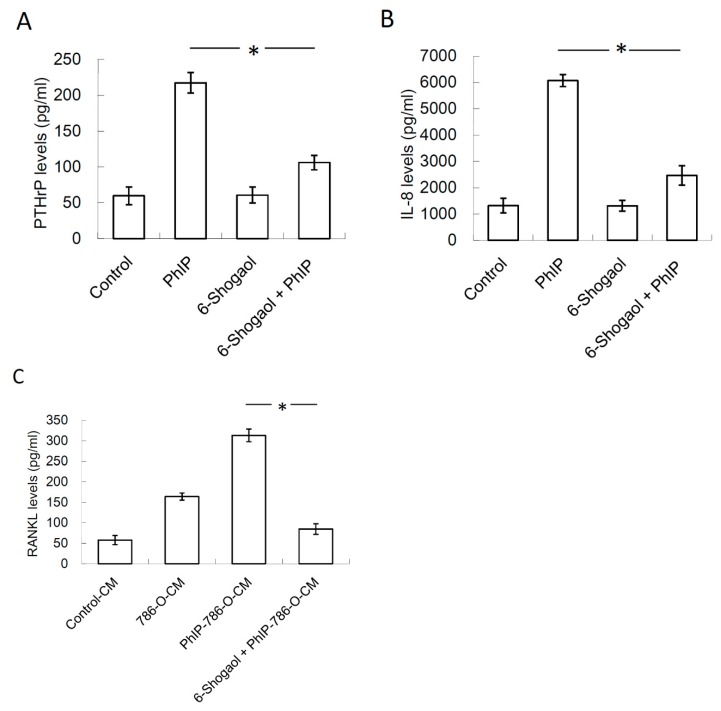
6-Shogaol decreased the effects of PhIP on PTHrP, IL-8, and RANKL expression. (**A**) The PTHrP levels in medium of 786-O. For assessing the level of PTHrP, 786-O cells were pre-treated with 6-shogaol for 1 h, and then with 20 μM PhIP for 6 h. After replacing fresh medium and another 24 h culture, the PTHrP levels in medium of 786-O were measured. (**B**) The IL-8 levels in medium of 786-O cells. For assessing the level of PTHrP, 786-O cells were pre-treated with 6-shogaol for 1 h, and then with 20 μM PhIP for 24 h. (**C**) The RANKL levels in medium of osteoclasts. For assessing RANKL, 786-O were pre-treated with 6-shogaol for 1 h, then incubated with 20 μM PhIP for 6 h. After replacing fresh medium and another 24 h of culturing, the culture medium of 786-O was harvested and then added to osteoblasts for a 24 h culture. Each value is the mean ± SD of three independent experiments. * *P* < 0.05, or significant difference between the control and test groups.

**Figure 6:**
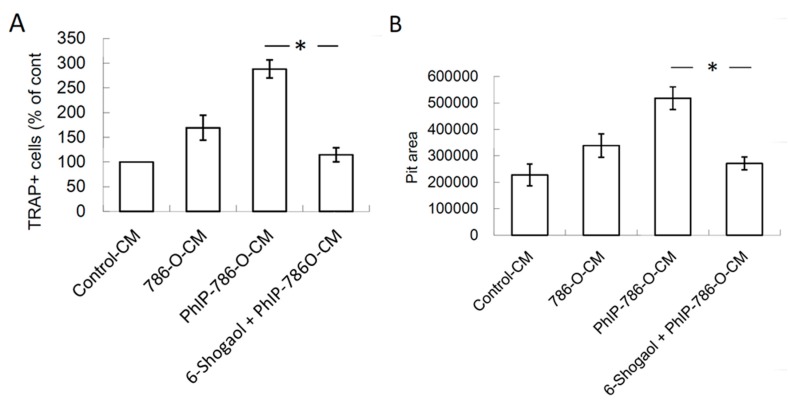
6-Shogaol decreased the effects of PhIP on in human 786-O renal cell carcinoma bone metastasis events. 786-O cells were pretreated with 6-shogaol for 1 h, and then incubated with 20 μM PhIP for another 6 h. After replacing fresh medium and following a 24 h culture, the culture medium was collected. Subsequently, CD14^+^ monocytes were cultured with medium with 20% of collected medium containing M-CSF and RANKL for 14–21 days. (**A**) The effect of 6-shogaol on osteoclastogenesis and (**B**) bone resorption. Each value is the mean ± SD of three independent experiments. * *P* < 0.05, or significant difference between the control and test groups.

The effects of 6-shogaol on PhIP-induced renal cell carcinoma bone metastasis was evaluated. PhIP induced PTHrP and IL-8 secretion in human 786-O renal cell carcinoma cells, which decreased by 2 μM 6-shogaol treatment (Figure 5A,B). Similarly, the PhIP-786-O-CM-treated 786-O cells mediated the RANKL up-regulation in osteoblasts (Figure 5C). In Figure 6A,B, osteoclastogenesis and bone resorption were significantly abrogated by the 6-shogaol treatment. The results revealed that 6-Shogaol suppressed PhIP-mediated bone resorption.

## 4. Discussion

The interactions between the osteoblast and osteoclast regulate the bone remodeling in osteolytic metastasis [43]. Previous studies have reported that PTHrP can stimulate osteoclastogenesis by increasing the RANKL expression and by reducing the OPG expression in osteoblasts [44,45]. In addition, RANKL induces the formation of mature osteoclasts in the presence of M-CSF [46,47]. By contrast, the interaction between OPG, which is a decoy receptor of RANKL and RANKL, decreases osteoclastogenesis [48], so increasing the ratio of RANKL/OPG results in osteoclastic bone resorption [44,45]. The present study shows that PhIP pre-treatment up-regulates the PTHrP expression in renal cell carcinoma 786-O. PhIP-CM-786-O enhances the M-SCF and RANKL expression and represses the OPG expression in osteoblasts. These results suggest that PhIP might be a risk factor for bone metastasis in RCC.

A previous study has reported that high levels of IL-8 enhance both osteoclastogenesis and bone resorption in RCC [49]. Furthermore, PTHrP enhances osteoclastogenesis through inducing osteoclast stimulatory factors, such as IL-8 [50,51]. Our results show that PhIP increases the IL-8 expression in human 786-O cells. Silencing PTHrP via siRNA abolishes the PhIP-mediated up-regulation of IL-8, suggesting that PTHrP is a major mediator involved in the stimulatory effect of PhIP on IL-8 production. Furthermore, PhIP enhances osteoclastogenesis and bone resorption activity via the CM of 786-O cells. Thus, the regulation of PTHrP/IL-8 and PhIP plays a key role in PhIP-induced osteoclastogenesis and bone resorption in RCC.

The prognosis of metastatic RCC is poor [27], and current treatments for bone metastases have limited efficacy, while some side effects decrease the quality of life of RCC patients [52,53]. Therefore, the development of new therapies is still an important issue [54]. 6-Shogaol inhibits breast cancer cell invasion by reducing the matrix metalloproteinase-9 expression through blocking nuclear factor-κB activated-migration in breast cancer [55]. However, PTHrP and transforming growth factor-β (TGF-β) promote mutual expression and form a vicious cycle in breast cancer [56].

As osteolytic bone metastases have been a major type in breast cancer and RCC [29,30], the effect of 6-Shogaol on bone metastasis and the potential mechanism was investigated in this study. Our results show that 6-shogaol exhibits effects that decrease the PTHrP expression in human 786-O renal cell carcinoma cells. Simultaneously, 6-shogaol also decreases IL-8 expression and then results in the inhibition of 786-O-mediated osteoclastogenesis and bone resorption. Moreover, 6-shogaol also decreases the PhIP-786-O-CM-induced RANKL expression in osteoblasts, suggesting that 6-shogaol might be a potential agent for preventing the aggravating effect of PhIP on renal cell carcinoma bone metastasis.

The cytotoxicity of 6-shogaol has been evaluated in several types of cells in previous studies in vitro and in vivo. In our previous study, the treatment of 80 μM 6-shogaol did not significantly affect the viability of a normal lung cell line [14]. In another study, the IC50 value of 6-shogaol on the normal colon and lung cell line was 43.91 and 36.65 μM, respectively [57]. This evidence suggests that 2 μM 6-shogaol did not significantly affect the viability of normal cells in vitro. The consumption of 2.0 g ginger and its constituents daily has low toxicity and high tolerability in animals and humans [58]. The oral intake of a single dose of red ginger suspension (2 g/15 mL) reached maximum plasma concentration of 6-shogaol at 453.40 ng/mL (approximately 1.6404 μM) in heathy volunteers [59]. Another study showed that the oral administration of 2 g ginger extract (containing 7.4 mg of 6-shogaol) and 6-shogaol could not be detected in plasma, suggesting that 6-shogaol is rapidly metabolized in the human body [60].

A recent report indicated that 6-shogaol-loaded novel micelles increased the oral bioavailability in a rat model [61]. This might be a potential strategy to further enhance the bioactivities of 6-shogaol for cancer treatment in the future. Apart from the direct oral administration of the ginger extract or pure 6-shogaol and its metabolites, a previous study demonstrated that ginger processing can increase the 6-shogaol content via the storage or drying of ginger rhizome. Therefore, old rhizomes contain significantly higher contents of 6-shogaol [62,63]. In addition, ginger processed via steam heating can further undergo conversion of 6-gingerol to 6-shogaol [64]. The constituents of 6-, 8-, and 10-shogaol significantly increase after processing ginger in an aqueous solution under microwaves [65,66]. Therefore, the investigation of the compositions of ginger via different types of food processing is an important issue and worthy of further determination in future studies.

**Figure 7:**
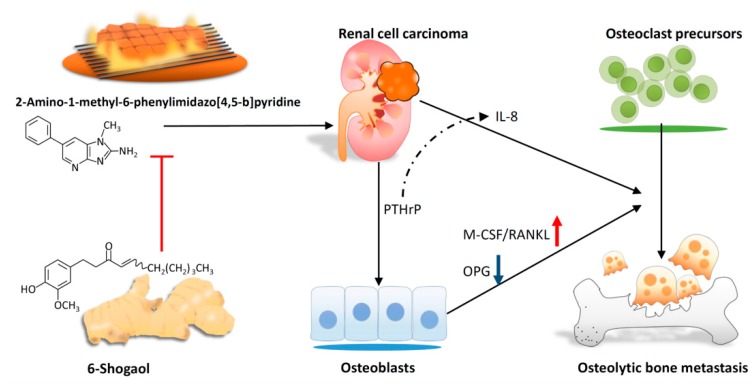
Scheme of proposed 6-shogaol-inhibited PhIP-induced human renal cell carcinoma bone metastasis.

The elimination all PhIP exposure might be difficult because PhIPs are widely used in modern life, so the development of novel strategies for preventing and treating bone metastasis is necessary. Although the dosages of PhIP and 6-shogaol in the in vitro experiments are not perfectly relevant to human exposure, this study is still the first to demonstrate that PTHrP produced by PhIP-exposed human 786-O renal cell carcinoma cells contributed to bone metastasis by increasing osteoclastogenesis. This is also the first study to reveal that 6-shogaol reverses PhIP-mediated bone resorption in an experimental RCC model in vitro (Figure 7).

## 5. Conclusions

In summary, this study firstly demonstrated that PhIP is a risk factor to induce osteoclastogenic activity and metastatic potential in human 786-O renal cell carcinoma cell line, while 6-Shogaol treatment reverses the PhIP-induced effect.
